# Radiographic analysis of specific morphometrics and patient-reported outcomes (PROMIS) for surgical repair of zones 2 and 3 fifth metatarsal fractures

**DOI:** 10.1186/s13018-021-02331-7

**Published:** 2021-03-22

**Authors:** Rusheel Nayak, Joshua Barrett, Milap S. Patel, Mauricio P. Barbosa, Anish R. Kadakia

**Affiliations:** 1 Department of Orthopaedic Surgery, Feinberg School of Medicine, Northwestern University, 676 North St. Clair Street #1350, Chicago, IL 60611 USA; 2 Clinica Orthobone, Rua Joaquim Floriano, 466, Conjunto 1.503 - Itaim Bibi, São Paulo, 04534-002 Brazil

**Keywords:** Jones fracture, Proximal fifth metatarsal fracture, Intramedullary screw fixation, Complications, PROMIS, Patient-reported outcomes, Physical function, Pain interference

## Abstract

**Background:**

Zones 2 and 3 fifth metatarsal fractures are often treated with intramedullary fixation due to an increased risk of nonunion. A previous 3-dimensional (3D) computerized tomography (CT) imaging study by our group determined that the screw should stop short of the bow of the metatarsal and be larger than the commonly used 4.5 millimeter (mm) screw. This study determines how these guidelines translate to operative outcomes, measured using Patient-Reported Outcomes Measurement Information System (PROMIS) Physical Function (PF) and Pain Interference (PI) surveys. Radiographic variables measuring the height of the medial longitudinal arch and degree of metatarsus adductus were also obtained to determine if these measurements had any effect on outcomes. And lastly, this study aimed to determine if morphologic differences between males and females affected surgical outcomes.

**Methods:**

We retrospectively identified 23 patients (14 male, 9 female) who met inclusion criteria. Eighteen patients completed PROMIS surveys. Preoperative PROMIS surveys were completed prior to surgery, rather than retroactively. Weightbearing radiographs were also obtained preoperatively to assist with surgical planning and postoperatively to assess interval healing. Correlation coefficients were calculated between PROMIS scores and repair characteristics (hardware characteristics [screw length and diameter] and radiographic measurements of specific morphometric features). *T* tests determined the relationship between repair characteristics, PROMIS scores, and incidence of operative complications. PROMIS scores and correlation coefficients were also stratified by gender.

**Results:**

The average screw length and diameter adhered to guidelines from our previous study. Preoperatively, mean PROMIS PI = 57.26±11.03 and PROMIS PF = 42.27±15.45 after injury. Postoperatively, PROMIS PI = 44.15±7.36 and PROMIS PF = 57.22±10.93. Patients with complications had significantly worse postoperative PROMIS PF scores (*p=0.*0151) and PROMIS PI scores (*p=*0.003) compared to patients without complications. Females had non-significantly worse preoperative and postoperative PROMIS scores compared to males and had a higher complication rate (33 percent versus 21 percent, respectively). Metatarsus adductus angle was shown to exhibit a significant moderate inverse relationship with postoperative PROMIS PF scores in the overall cohort (*r=−*0.478; *p*=0.045). Metatarsus adductus angle (*r=−*0.606; *p*=0.008), lateral talo-1st metatarsal angle (*r=−*0.592; *p*=0.01), and medial cuneiform height (*r=−*0.529; *p*=0.024) demonstrated significant inverse relationships with change in PROMIS PF scores for the overall cohort. Within the male subcohort, significant relationships were found between the change in PROMIS PF and metatarsus adductus angle (*r=−*0.7526; *p*=0.005), lateral talo-1st metatarsal angle (*r=−*0.7539; *p*=0.005), and medial cuneiform height (*r=−*0.627; *p*=0.029).

**Conclusion:**

Patients treated according to guidelines from our prior study achieved satisfactory patient reported and radiographic outcomes. Screws larger than 4.5mm did not lead to hardware complications, including screw failure, iatrogenic fractures, or cortical blowouts. Females had non-significantly lower preoperative and postoperative PROMIS scores and were more likely to suffer complications compared to males. Patients with complications, higher arched feet, or greater metatarsus adductus angles had worse functional outcomes. Future studies should better characterize whether patients with excessive lateral column loading benefit from an off-loading cavus orthotic or plantar-lateral plating.

## Background

Proximal fifth metatarsal fractures are among the most common fractures of the foot and account for over 50 percent of metatarsal fractures in the general population [1, 2]. Lawrence and Botte developed a classification for proximal fifth metatarsal fractures based on location [3]. Zone 1 fractures, or proximal cancellous tuberosity avulsion fractures, are treated nonoperatively with excellent outcomes [4]. Zone 2 fractures, commonly called Jones fractures, extend from the cancellous tuberosity to the articulation between the fourth and fifth metatarsal [4, 5]. This area, at the metaphyseal-diaphyseal junction, has been described as a watershed zone due to poor blood supply; consequently, fractures treated nonoperatively in this zone have an increased risk of nonunion [4]. Evidence suggests that intramedullary compression screw fixation is indicated in competitive athletes and can decrease healing time and rates of nonunion in the general population [6–8]. Similarly, a trial of conservative management may be attempted for acute zone 3, or proximal diaphyseal, fractures, but operative treatment may be required if there are clinical or radiographic signs of delayed union or nonunion [9, 10].

Choosing the right screw is important to ensure the correct fit and optimal compression at the fracture site. However, the challenge in picking the optimal screw stems from fitting a straight screw into a poorly vascularized bone with lateroplantar curvature [11, 12]. Using an excessively long screw can result in straightening of the bone, lateral cortical gapping, and perforation of the medial cortex. Using a larger diameter screw than indicated can result in iatrogenic fracture or cortical blowout, while a smaller screw can result in decreased thread purchase and fracture stability. A screw of incorrect length or diameter increases the risk of nonunion, delayed union, hardware failure, and refracture [13–16]. Multiple studies have utilized cadaveric analyses [16–20] and computerized tomography [11, 12, 21] to gain a better understanding of the anatomy of the fifth metatarsal to determine the optimal screw length and diameter for intramedullary fixation of zones 2 and 3 fractures.

A recent study by this group (Ochenjele et al. [12]) used 3D computerized tomography to more precisely determine the anatomy of the proximal fifth metatarsal and inform operative management. The study determined that the optimal screw length should be no longer than the straight length segment, defined as the distance from the base of the metatarsal to the shaft of the curvature. This value can be estimated for each patient by subtracting 10 percent of the full metatarsal length on the anteroposterior (AP) or oblique view and then calculating 68 percent of the resulting value. The optimal screw diameter can be estimated by measuring the coronal medullary canal diameter at the bow of the fifth metatarsal on the AP projection. It was determined that the vast majority of patients would likely require screws larger than 4.5 mm to achieve adequate endosteal fixation.

The purpose of this study is to determine how these new advancements in anatomical knowledge of the fifth metatarsal translate to operative outcomes. Operative outcomes will be measured using the Patient-Reported Outcomes Measurement Information System (PROMIS). PROMIS is a National Institutes of Health (NIH) funded computerized adaptive testing (CAT) system that derives patient-reported outcome (PRO) scores. PRO scores are becoming an increasingly important aspect of orthopedic patient care, as they provide patient input that can be used to complement clinical and radiographic evidence of treatment outcomes. PROMIS was recently validated as an effective tool in predicting operative outcomes in foot and ankle surgery [22]. The study also aims to assess how foot morphology impacts the incidence of complications and PROMIS scores. And lastly, DeSandis et al. [11] found that fifth metatarsal morphology—specifically metatarsal length, canal diameter, and apex height—differ significantly by gender. This study aims to determine whether morphologic differences between males and females affect operative outcomes.

## Methods

Following appropriate institutional board review approval, all patients who underwent an open reduction and internal fixation (ORIF) for a proximal fifth metatarsal fracture at our institution between November 2013 and November 2016 were reviewed. Patients were excluded if they had history of prior proximal fifth metatarsal fracture surgery or injury to the fifth metatarsal, Charcot arthropathy, or any midfoot/forefoot conditions that disrupted the normative anatomy of the fifth metatarsal in relation to the rest of the foot. All patients underwent operative fixation using fully threaded cannulated screws. A query of our institutional data warehouse yielded 24 patients who met inclusion criteria. Twenty-three patients were included in the study following one exclusion due to concurrent bilateral procedures. All included patient records were qualitatively reviewed to confirm diagnosis of a fracture of the proximal fifth metatarsal of zones 2 or 3. There were 21 zone 2 fractures and two zone 3 fractures in our cohort. Twenty-one patients had baseline PROMIS Physical Function data and 20 have baseline PROMIS Pain Interference data; 18 had postoperative follow-up PROMIS data.

**Fig. 1:**
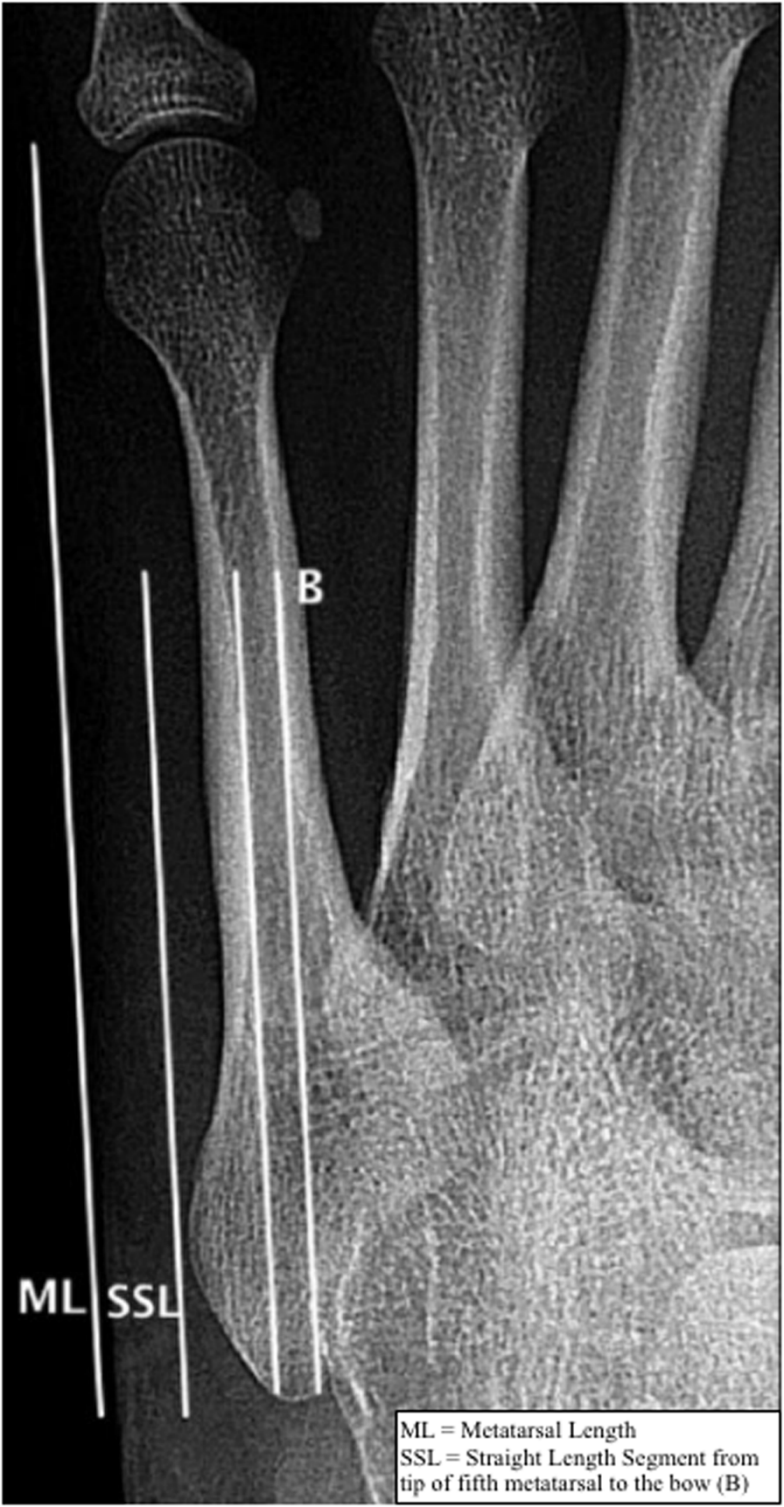
Radiographic measurements pertaining to the fifth metatarsal. AP radiograph demonstrating how measurements pertaining to the fifth metatarsal were performed. The medullary canal diameter was measured at the bow of the metatarsal (labeled B)

All 23 patients included were determined to have standard weightbearing foot series radiographs (AP, medial oblique, lateral). Each of the radiographs utilized for our study were exported from picture archiving and communication system (PACS, General Electric Healthcare, Barrington, Illinois) to the Centricity Universal Viewer (General Electric Healthcare, Barrington, Illinois). A fellowship trained foot and ankle surgeon (M.B.) assessed all radiographs for image quality and conducted relevant measurements of the ipsilateral foot. Radiographic measurements used in this study included calcaneal pitch angle, metatarsus adductus angle, AP talo-1st metatarsal angle, lateral talo-1st metatarsal angle (Meary’s angle), and medial cuneiform height. Length of the fifth metatarsal, width of the medullary canal at the bow, and distance of fracture from the proximal tip of the fifth metatarsal were measured as well (Fig. 1).

The patients included in this study were asked to complete preoperative and postoperative PROMIS Physical Function v1.2 (PF) and Pain Interference v1.1 (PI) surveys. PROMIS PF measures self-reported ability to perform functional activities, with a higher PF score indicating higher physical function. PROMIS PI measures how pain impacts the patient’s daily life, with higher PI scores indicating higher pain levels. The PROMIS CAT algorithm for each domain produces a T-score standardized to the general US population with a range of 0-100, a mean of 50, and a standard deviation of ten.

Of the 23 patients in our study, 14 were males and nine were females. The mean age of our study population was 38 ± 17 years. Twelve fractures occurred in the right fifth metatarsal and 11 fractures occurred in the left metatarsal. The average body mass index (BMI, kilogram/meter^2^) of the cohort was 30.76. One patient was a current smoker and there were two diabetic patients in this cohort. Table 1 shows patient demographics, mechanism of injury, and any operative complications. Six patients—three of fourteen males and three of nine females—had operative complications (24 percent). Characteristics of these patients, including pre-existing comorbidities, are shown in Table 2.

**Table 1:** Patient demographics, mechanism of injury, surgical complications

Patient	Age	Gender	BMI	Mechanism	Sport	Screw length (mm)	Screw diameter (mm)	Complication
1	66	Female	27.24	Add-invert	No	40	5.5	No
2	65	Female	26.08	Add-invert	No	40	5.5	No
3	48	Male	35.49	Add-invert	No	40	5.5	Yes
4	70	Female	21.63	Add-invert	No	40	5.5	No
5	33	Female	44.41	Stress	No	40	5.5	Yes
6^a^	28	Male	26.04	Stress	No	50	4.75	Yes
7	27	Female	57.59	Add-invert	No	40	5.5	No
8	24	Male	22.60	Add-invert	No	35	5.5	No
9	61	Female	37.55	Hyper-extension	No	40	5.5	No
10	42	Male	27.68	Add-invert	No	35	5.5	No
11^a^	19	Male	22.50	Stress	Soccer	50	5.5	No
12	25	Male	26.87	Add-invert	No	45	5.5	No
13	20	Male	26.88	Add-invert	Football	45	5.5	No
14	25	Male	23.10	Add-invert	Basketball	40	4.75	No
15	31	Male	29.28	Add-invert	No	40	5.5	No
16	54	Female	48.65	Stress	No	40	5.5	Yes
17	20	Male	31.76	Add-invert	Basketball	45	5.5	No
18	18	Female	21.19	Add-invert	Ballet	40	5.5	No
19	63	Male	32.47	Stress	Run	35	5.5	Yes
20	44	Male	32.43	Stress	No	45	5.5	No
21	36	Male	22.22	Add-invert	Work out	55	4.75	No
22	28	Male	31.25	Add-invert	Run	45	5.5	No
23	28	Female	32.47	Add-invert	No	45	5.5	Yes

^a^Zone 3 fracture

Zone 2 unless otherwise noted; *Add-invert* adduction-inversion

**Table 2:** Characteristics of complications

Patient	Comorbidities	Complication	Treatment	Outcome
3	BMI 35.49 (class II obesity), diabetes mellitus 2	Re-fracture at 10 months	Hardware removal, *ORIF, and bone graft at an outside hospital at 11 months	Lost to follow-up
5	BMI 44.41 (class III obesity)	Re-fracture at 4 months	Revision surgery with plate, bone graft, **BMC at 6 months	Union at 6 months post-revision
6	None	Re-fracture at 6 months	Conservative management	Healing at 12 months
16	BMI 48.65 (class III obesity), chronically low vitamin D levels,	Persistent non-union	Revision ORIF with plating and bone grafting at 7 months	Stable hardware, fracture healed at 20 months post-revision
19	BMI 32.47 (class I obesity), psoriatic arthritis, congenital varus deformity status-post calcaneal osteotomy	Persistent non-union	Conservative management	Stable hardware and stable, asymptomatic nonunion at 12 months
23	BMI 32.47 (class I obesity), osteoporosis secondary to systemic steroids for severe asthma	Persistent non-union	Conservative management	Fracture nearly closed at 9 months, patient lost to follow up thereafter

*ORIF open reduction and internal fixation; **BMC bone marrow concentrate

### Statistical analysis

Statistical analysis was performed using the Stata 10 Statistical Software (StataCorp, College Station, Texas). *T* tests (alpha = 0.05) were performed to test for the presence of a relationship between repair characteristics and incidence of any operative complications. Correlation coefficients (*r*) were calculated between PROMIS scores at postoperative follow-up and repair characteristics (radiographic measurements, screw length, screw diameter). Correlation coefficients were then calculated comparing change in PROMIS scores from preoperative baseline and repair characteristics. Results were also stratified by gender, where appropriate. Correlation coefficients between PROMIS scores and screw length and screw diameter were not stratified by gender due to lack of variation and small sample sizes within the subcohorts.

## Results

Table 3 describes screw characteristics used for fracture repair in this population, subdivided by gender. The mean screw length was 42.17 ± 4.96 mm with a minimum length of 35 mm and a maximum length of 55 mm. The mean screw length used in males (43.21 ± 6.10 mm) was larger than the mean screw length used in females (40.56 ± 1.67 mm). The mean screw diameter was 5.40 ± 0.28 mm with a minimum diameter of 4.7 mm and a maximum of 5.5 mm. The mean screw diameter used in males (5.34 ± 0.33 mm) was marginally smaller than the mean screw length used in females (5.5 ± 0.0 mm). There were no significant differences in screw length or diameter by gender. Ratios of repair characteristics are also listed in Table 3.

**Table 3:** Morphological characteristics

Characteristic	Mean	Std. deviation	Minimum	Maximum
**Screw length (mm)**	**42.17**	**4.96**	**35**	**55**
Screw length (female)	40.56	1.67	40	55
Screw length (male)	43.21	6.10	35	55
**Screw diameter (mm)**	**5.40**	**0.28**	**4.7**	**5.5**
Screw diameter (female)	5.50	0	5.5	5.5
Screw diameter (male)	5.34	0.33	4.7	5.5
**Screw length (mm)/distance from screw insertion to fracture (mm)**	**1.89**	**0.3**	**1.52**	**2.86**
Above ratio (female)	2.01	0.42	1.5	2.9
Above ratio (male)	1.81	0.20	1.5	2.2
**Screw length (mm)/distance from fracture to fifth metatarsal bow (mm)**	**0.78**	**0.09**	**0.59**	**1.02**
Above ratio (female)	0.81	0.09	0.74	1.02
Above ratio (male)	0.75	0.09	0.59	0.89
**Screw diameter/medullary canal diameter (mm)**	**0.82**	**0.15**	**0.55**	**1.17**
Above ratio (female)	0.82	0.17	0.7	1.2
Above ratio (male)	0.83	0.16	0.6	1.1

Table 4 shows mean preoperative and postoperative PROMIS scores for the Physical Function and Pain Interference domains. Postoperative scores (PF: 57.38 ± 11.16; PI: 44.15 ± 7.36) were statistically significantly different from preoperative scores (PF: 42.27 ± 15.45; PI: 57.26 ± 11.03) for the overall cohort (*p<*0.05). Postoperative scores (PF: 59.47 ± 11.00; PI: 43.49 ± 7.52) were statistically significant compared to preoperative scores for the (PF: 44.82 ± 17.01; PI: 54.73 ± 10.41) male cohort (*p<*0.05). Postoperative PROMIS PI scores (45.46 ± 7.54) were statistically significant compared to preoperative scores (61.96 ± 11.36) for female cohort (*p<*0.05). Postoperative PROMIS PF scores (53.22 ± 11.26) trended toward significance compared to preoperative PROMIS PF scores (38.13 ± 12.41) for female cohort, but were ultimately not significant (*p>*0.05). Table 5 shows mean change in PROMIS scores postoperatively compared to preoperative baseline after injury. There were no significant differences in preoperative, postoperative, or change in PROMIS scores between genders.

**Table 4:** Mean PROMIS scores

	Preoperative PF	Postoperative PF	Preoperative PI	Postoperative PI
**Overall Cohort**	**42.27 ± 15.45**	**57.38 ± 11.16**	**57.26 ± 11.03**	**44.15 ± 7.36**
Female	38.13 ± 12.41	53.22 ± 11.26	61.96 ± 11.36	45.46 ± 7.54
Male	44.82 ± 17.01	59.47 ± 11.00	54.73 ± 10.41	43.49 ± 7.52

Postoperative PROMIS PF and PROMIS PI scores statistically significant compared to preoperative scores for the overall cohort and male cohort. Postoperative PROMIS PI scores statistically significant compared to preoperative scores for female cohort. Postoperative PROMIS PF scores not statistically significant compared to preoperative scores for female cohort (*a* = 0.05)

**Table 5:** Mean change in PROMIS scores

	PROMIS PF	PROMIS PI
**Overall change**	**+13.98**	**11.99**
Female	+11.77	13.08
Male	+15.08	11.54

Table 6 shows morphometric measurements (calcaneal pitch angle, lateral talo-1st metatarsal angle, medial cuneiform height, AP talo-1st metatarsal angle, and metatarsus adductus angle) for each patient in the cohort. Table 7 shows mean morphometric measurements, separated by gender. No significant difference was found in any morphometric measurements when comparing males and females. Table 8 shows correlation coefficients (*r*) between repair characteristics and postoperative PROMIS PF and PI scores. Metatarsus adductus angle was shown to exhibit a significant (*p =* 0.045) moderate inverse relationship (*r =* −0.478) with postoperative PROMIS PF scores. No other repair characteristic showed a significant correlation with postoperative PROMIS scores.

**Table 6:** Morphometric measurements

Patient	Calcaneal pitch angle	Talo-1st metatarsal angle (lateral)	Medial cuneiform height	Talo-1st metatarsal angle (AP)	Metatarsus adductus angle
**1**	11	−16	15	16	16
**2**	25	−7	20	5	18
**3**	30	−13	25	−1	27
**4**	24	1	24	−6	21
**5**	22	−5	26	−1	20
**6**	18	−5	22	4	22
**7**	24	−9	21	1	23
**8**	25	−4	19	10	20
**9**	32	6	23	12	7
**10**	27	−4	23	13	15
**11**	23	−9	19	5	20
**12**	24	−12	17	5	8
**13**	30	13	31	−11	27
**14**	27	1	26	1	25
**15**	24	1	28	−9	20
**16**	16	9	25	−18	26
**17**	16	1	24	1	21
**18**	18	−12	14	12	20
**19**	32	−3	29	−19	10
**20**	20	−11	17	3	28
**21**	37	6	30	−3	29
**22**	22	3	27	−7	29
**23**	12	−18	11	5	30

**Table 7:** Mean morphologic characteristics

	Calcaneal pitch angle	Talo-1st metatarsal angle (lateral)	Medial cuneiform height	Talo-1st metatarsal angle (AP)	Metatarsus adductus angle
**Overall cohort**	**23.4 ± 6.5**	**−3.8 ± 8.2**	**22.4 ± 5.3**	**.79 ± 9.3**	**21.0 ± 6.5**
Female	20.4 ± 6.8	**−**5.7 ± 9.4	19.9 ± 5.3	2.9 ± 10.5	20.1 ± 6.5
Male	25.4 ± 5.7	**−**2.6 ± 7.4	24.1 ± 4.7	**−**0.57 ± 8.6	21.5 ± 6.7

**Table 8:** Correlation of morphological characteristics and postoperative PROMIS PF and PI

Repair characteristics	PROMIS PF [***r*** (***p***)]	PROMIS PI [***r*** (***p***)]
**Calcaneal pitch angle**	**−0.009 (0.973)**	**−0.161 (0.524)**
Female	**−**0.0641 (0.903)	**−**0.538 (0.270)
Male	**−**0.217 (0.498)	0.0566 (0.861)
**Metatarsus adductus angle**	**−0.478 (0.045)**^**a**^	**0.218 (0.385)**
Female	**−**0.541 (0.27)	0.063 (0.905)
Male	**−**0.4924 (0.104)	0.260 (0.414)
**Talo-1st metatarsal angle (lateral)**	**−0.22 (0.381)**	**−0.058 (0.819)**
Female	**−**0.6130 (0.196)	0.314 (0.545)
Male	**−**0.117 (0.717)	**−**0.227 (0.479)
**Talo-1st metatarsal angle (AP)**	**0.357 (0.145)**	**0.01 (0.97)**
Female	0.714 (0.111)	**−**0.303 (0.559)
Male	0.205 (0.523)	0.182 (0.571)
**Medial cuneiform height (mm)**	**−0.411 (0.09)**	**0.092 (0.716)**
Female	**−**0.732 (0.098)	0.351 (0.495)
Male	**−**0.486 (0.110)	0.0487 (0.885)
**Screw size (mm)**	**−0.01 (0.969)**	**−0.102 (0.687)**
Female		
Male		
**Screw diameter (mm)**	**0.2656 (.287)**	**−0.090 (.722)**
Female		
Male		

^a^Statistically significant (*a* = 0.05); [*r* (*p*)] = [correlation (*p* value)]

Table 9 shows correlation coefficients (*r*) between repair characteristics and the change in PROMIS PF and PI scores preoperatively and postoperatively. Significant inverse relationships were found between the delta values for the PROMIS PF domain and the metatarsus adductus angle (*r=−*0.606; *p*=0.008), lateral talo-1st metatarsal angle (*r=*−0.592; *p*=0.01), and medial cuneiform height (*r*=−0.529; *p*=0.024). These relationships can be described as moderate inverse correlations in which a smaller metatarsus adductus angle, lateral talo-1st metatarsal angle, and medial cuneiform heights correlate with statistically significantly greater change in patient-reported physical function scores. Within the male subcohort, significant relationships were found between the delta values for PROMIS PF and metatarsus adductus angle (*r =* −0.7526; *p* = 0.005), lateral talo-1st metatarsal angle (*r* = −0.7539; *p* = 0.005), and medial cuneiform height (*r* = −0.627; *p* = 0.029).

**Table 9:** Correlation of morphological characteristics and change in PROMIS PF and PI

Morphological characteristics	PROMIS PF [***r*** (***p***)]	PROMIS PI [***r*** (***p***)]
**Calcaneal pitch angle**	**−0.120 (0.636)**	**0.284 (0.270)**
Female	0.132 (0.804)	**−**0.243 (0.694)
Male	**−**0.307 (0.332)	0.452 (0.140)
**Metatarsus adductus angle**	**−0.606 (0.008)**^**a**^	**−0.383 (.129)**
Female	**−**0.013 (0.981)	**−**0.283 (0.645)
Male	**−**0.7526 (0.005)^**a**^	0.513 (0.088)
**Talo-1st metatarsal angle (lateral)**	**−0.592 (0.01)**^**a**^	**0.190 (0.468)**
Female	**−**0.384 (0.452)	0.232 (0.708)
Male	**−**0.7539 (0.005)^**a**^	0.166 (0.607)
**Talo-1st metatarsal angle (AP)**	**−0.349 (0.156)**	**−0.039 (0.88)**
Female	0.408 (0.422)	**−**0.0450 (0.943)
Male	0.337 (0.283)	**−**0.041 (0.900)
**Medial cuneiform height (mm)**	**−0.529 (0.024)**^**a**^	**0.253 (0.327)**
Female	**−**0.552 (0.256)	**−**0.0717 (0.909)
Male	**−**0.627 (0.029)^**a**^	0.344 (0.274)
**Screw length (mm)**	**−0.123 (0.627)**	**−0.056 (0.831)**
Female		
Male		
**Screw diameter (mm)**	**0.1701 (0.500)**	**−0.201 (.438)**
Female		
Male		

^a^Statistically significant (*a* = 0.05); [*r* (*p*)] = [correlation (*p* value)]

*T* tests were used to determine if there were statistically significant differences in PROMIS scores or radiographic characteristics of those who had uncomplicated recoveries versus those who experienced complications. The results are shown in Tables 10 and 11. Postoperative PROMIS PF (*p =* 0.0151) and postoperative PROMIS PI (*p =* 0.003) scores were shown to be statistically significantly different in patients who did not have complications compared to patients who had complications. BMI approached significance (*p =* 0.0682). Tables 10 and 11 show descriptive statistics of PROMIS scores and radiographic characteristics by gender. Statistical analysis was not conducted due to a limited sample size within these subcohorts.

**Table 10:** Mean PROMIS scores and surgical complications

	No complications	Complications	***p*** value
**Preoperative PROMIS PF**	**42.27 ± 15.45**	**32.24 ± 8.43**	**0.1379**
Female	37.20 ± 14.43	40.90 ± 3.96	
Male	50.33 ± 15.42	26.47 ± 3.05	
**Postoperative PROMIS PF**	**57.22 ± 10.93**	**47.54 ± 10.07**	0.0151*
Female	57.43 ± 11.03	44.80 ± 7.50	
Male	62.83 ± 8.63	49.4 ± 12.74	
**Preoperative PROMIS PI**	**57.26 ± 11.03**	**63.56 ± 7.08**	**0.145**
Female	59.26 ± 12.66	68.70 ± 2.55	
Male	53.11 ± 10.95	60.1 ± 7.29	
**Postoperative PROMIS PI**	**44.15 ± 7.36**	**51.80 ± 7.79**	0.003*
Female	41.55 ± 5.70	53.30 ± 1.56	
Male	41.06 ± 4.69	50.80 ± 10.78	
**Change PROMIS PF**	**+15.47**	**+15.30**	**0.857**
Female	+15.7	+3.9	
Male	+12.48	+22.90	
**Change PROMIS PI**	−**13.02**	−**11.54**	**0.954**
Female	−11.53	−15.40	
Male	−12.27	−9.33	

^*^statistically significant (*a* = 0.05)

**Table 11:** Morphological characteristics and surgical complications

Characteristics	No complication	Surgical complication	***p*** value
**BMI (kg/m**^**2**^**)**	**28.70 ± 8.71**	**36.59 ± 8.41**	**.0682**
Female	31.9 ± 14.0	41.8 ± 8.38	
Male	27.0 ± 3.91	31.3 ± 4.8	
**Calcaneal pitch angle**	**24.06 ± 6.02**	**21.67 ± 7.94**	**.4487**
Female	22.3 ± 7.12	16.67 ± 5.03	
Male	25.0 ± 5.46	26.7 ± 7.6	
**Metatarsus adductus angle**	**20.41 ± 6.45**	**22.50 ± 7.09**	**.5131**
Female	17.5 ± 5.68	25.33 ± 5.03	
Male	22.0 ± 6.53	19.6 ± 8.7	
**Talo-1st metatarsal angle (lateral)**	−**3.06 ± 7.92**	−**5.83 ± 9.26**	**.4870**
Female	−6.17 ± 8.23	−4.67 ± 13.50	
Male	−1.36 ± 7.58	−7.0 ± 5.3	
**Talo-1st metatarsal angle (AP)**	**2.82 ± 8.14**	−**5.00 ± 10.75**	**.0762**
Female	6.67 ± 8.24	−4.67 ± 11.93	
Male	0.72 ± 7.64	−5.3 ± 12.1	
**Medial cuneiform height (mm)**	**22.24 ± 5.11**	**23.00 ± 6.29**	**.7690**
Female	19.50 ± 4.13	20.67 ± 8.39	
Male	23.72 ± 5.12	25.3 ± 3.5	
**Screw length (mm)**	**42.35 ± 5.04**	**41.67 ± 5.16**	**.7783**
Female	40.0 ± 0.0	41.67 ± 2.88	
Male	43.63 ± 5.95	41.7 ± 7.6	
**Screw diameter (mm)**	**5.41 ± 0.27**	**5.38 ± 0.29**	**.8624**
Female	5.5 ± 0.0	5.5 ± 0.0	
Male	5.35 ± 0.32	5.3 ± 0.4	

## Discussion

First described by Sir Robert Jones [23] in 1902, proximal fifth metatarsal fractures have been a focus of study due to the poor healing potential of many of these fractures. Lawrence and Botte [3] classified proximal fifth metatarsal fractures by location, with evidence suggesting that internal fixation of zones 2 and 3 fractures leads to superior results compared to conservative management [8, 24]. However, controversy still exists on the mode of fixation required for optimal results. Current management techniques have shown the efficacy of intramedullary screw fixation [6–8] although the optimal characteristics of the screw (length and diameter) are still debated. This study aims to determine how better anatomical understanding translates to patient-reported outcomes using PROMIS CAT surveys. Ho et al. recently validated PROMIS, but did not apply PROMIS to individual procedures [22]. To our knowledge, only Carney et al. [6] have looked at PROMIS as it relates to intramedullary fixation of the proximal fifth metatarsal. We are the first group to correlate radiographic variables with PROMIS scores pre- and postoperatively and to stratify these results by gender.

PROMIS PF and PROMIS PI improved significantly after surgery. While Carney et al. [6] did not show specific PROMIS scores in their abstract, they noted improvement in PROMIS10 scores (a global PROMIS domain) at 12 months. In addition, they found that patients had significantly improved visual analog scores (VAS) for pain, foot and ankle ability measure (FAAM) scores, patient acceptable symptom state (PASS) at 6 months postoperatively. Adhikari et al. [25] also demonstrated significant improvement in VAS pain after intramedullary screw fixation. While these studies used different PRO measures, these PRO scales have been shown to correlate well with changes in PROMIS scores [26–28]. Therefore, it is clear that patients who undergo intramedullary fixation of the proximal fifth metatarsal improve after surgery.

Patients with complications had higher levels of pain and lower functionality both preoperatively and postoperatively when compared to patients who did not have complications. While there was no statistically significant difference in preoperative PROMIS scores between those with complications and those without complications, patients who had complications had significantly lower postoperative PROMIS PF and PROMIS PI scores (*p = 0.*0151 and *p* = 0.003, respectively). However, the average improvement in scores from baseline in patients with complications was similar to patients without complications. Female patients had non-significantly lower physical function and higher pain preoperatively and postoperatively. In addition, a higher percentage of females (3 out of 9, 33 percent) had complications compared to the percentage of males (3 out of 14, 21 percent). These findings are similar to Cakir et al. [1], who found that AOFAS scores were significantly lower in females after metatarsal fracture.

Postoperative PROMIS scores were correlated with radiographic characteristics. Metatarsus adductus angle was found to have a moderate inverse correlation with postoperative PROMIS PF scores (*r* = −0.478, *p* = 0.045) and with change in PROMIS PF scores from baseline (*r *= −0.606; *p *= 0.008). This indicates that larger metatarsus adductus angles were correlated with significantly lower postoperative functional scores and lower change in functional scores from baseline. These findings are consistent with Yoho et al. [29], who found that every degree increase in metatarsus aductus increased bone healing time by 1.23 days. Lower functionality could be related to anatomy, due to continuous increased lateral load displacement. It is important to note, however, that metatarsus adductus angles were not significantly different in those who had uncomplicated recoveries versus those who had operative complications.

In addition, lateral talo-1st metatarsal angle (*r* = −0.592; *p* = 0.01), and medial cuneiform height were found to have a significant moderate inverse correlation with change in PROMIS PF scores from baseline. These relationships were also found within the male subcohort [lateral talo-1st metatarsal angle (*r =* −0.7539, *p *= 0.005); medial cuneiform height (*r* = −0.627, *p* = 0.029)]. Lateral talo-1st metatarsal angle and medial cuneiform height are measures of the medial longitudinal arch, indicating that a higher arch and midfoot adduction correlate with smaller postoperative improvement in functionality. Pes cavus is a well-established risk factor for proximal fifth metatarsal fractures due to persistent overloading of the lateral column [30–32]. Our study shows that patients with higher arches do not recover functionality as well as patients with flatter longitudinal arches, perhaps due to increased stresses on the lateral column during the recovery period and postoperatively. However, none of these radiographic measurements were significantly associated with operative complications.

The average proximal fifth metatarsal medullary canal diameter measured on AP radiograph was 6.6 ± 1.3 mm, indicating that a 4.0- or 4.5-mm screw would not be of sufficient size to ensure endosteal fixation. The median and mode screw diameter used in our study was 5.5 mm. The average screw diameter was 82 percent of the average medullary canal diameter. There were no cases of screw failure or cortical blowout, despite using screws larger than the 4.5 mm screw that was historically treated as the “gold standard” in proximal fifth metatarsal fracture surgery. Screw diameter was not significantly different in patients who had uncomplicated recoveries versus those who had complications. Numerous studies have found that that the largest screw diameter possible should be used to ensure optimal endosteal fixation [11, 12, 15, 19, 21, 33]. While this understanding stems largely from cadaveric or computerized tomography studies, Porter et al. [34] compared 5.5 mm and 4.5 mm screws in athletes. Similar to our study, Porter et al. [34] demonstrated the clinical efficacy of larger screws; however, they were unable to show a significant difference in outcomes over the 4.5 mm screw.

Ochenjele et al. [12] determined the optimal screw length corresponded with the straight length segment, or distance from the tip of the metatarsal base to the bow. This was determined on radiograph by subtracting 10 percent from the length of the fifth metatarsal and calculating 68 percent of the resulting value. In the average patient, this corresponded to a 40-mm screw. In this study, 47 percent (*n* = 11) received a 40 mm screw, with 39 percent (*n* = 9) receiving a screw longer than 40 mm. On average, the screws used in our study corresponded to 53.5 percent of the fifth metatarsal length or 78 percent of the straight length segment. The average screw length was nearly twice as long as the distance of the fracture from the proximal head of the fifth metatarsal, with a ratio of 1.89. This indicates that the screw provided optimal compression and support without exceeding the maximum recommended length. Screw length was not significantly different in patients who had uneventful recoveries compared to those who experienced complications. Similarly, screw length did not correlate significantly with postoperative PROMIS scores or changes in PROMIS scores from baseline.

Our study found that patients with larger metatarsus adductus angles and cavovarus radiological measurements (lateral talo-1st metatarsal angle and medial cuneiform height) correlated with worse functional outcomes after intramedullary fixation, likely due to lateral column overloading. While these radiological measurements were not associated with a higher rate of operative complications, it is possible that these patients may benefit from a plantar-lateral plate, rather than intramedullary screw, to offset the varus forces created by inherent foot anatomy. In their cadaveric study, Duplantier et al. [35] found that plantar-lateral plating provides better resistance to the forces placed on the proximal fifth metatarsal compared to intramedullary fixation. In addition, Bernstein et al. [36] found that athletes who experience repetitive lateral column loading had good operative outcomes after low-profile plating. While a plating technique requires a larger incision and carries the risk of disrupting blood supply to an already tenuously supplied region, plating has been shown to improve direct resistance of tensile forces could lead to improved functionality in carefully selected patients.

There are a few relevant limitations to this study. First, this study had a relatively small sample size, with *n* = 23 patients included in the study and *n* = 18 with completed PROMIS surveys. It is possible that the small sample size, combined with limited variance in certain variables such as screw diameter, resulted in type I error and less power to detect meaningful relationships between variables. As such, undetected relationships may have been more readily apparent with a larger sample. Second, it is possible that our cohort was affected by selection bias. Patients who underwent successful fifth metatarsal fracture surgeries are more likely to be lost to follow-up, potentially skewing results. In our dataset, five out of 23 patients (22%) did not complete follow-up PROMIS surveys. Further studies with larger sample sizes and greater time to follow-up are needed to better characterize how screw characteristics, specifically screw diameter, affect operative outcomes.

## Conclusion

In conclusion, patients who have proximal fifth metatarsal fracture have satisfactory operative outcomes, as measured by PROMIS PI and PROMIS PF scores. This study corroborates the clinical utility of larger diameter screws and the importance of the screw length stopping short of the bow of the metatarsal. Unsurprisingly, patients who had complications had significantly lower postoperative PROMIS PF scores compared to those without complications. Females had non-significantly lower preoperative and postoperative PROMIS scores and were more likely to suffer complications compared to males. Larger metatarsus adductus angles and radiological findings indicative of higher arches (lateral talo-1st metatarsal angle and medial cuneiform height) correlate with a smaller improvement in functionality after operative repair, especially in males. Future studies are required to determine whether patients with anatomy suggestive of excessive lateral column loading may benefit from an off loading cavus orthotic or plantar plating, rather than intramedullary screw fixation.

## Data Availability

The datasets generated and/or analyzed during the current study are not publicly available but are available from the corresponding author on reasonable request.

## Abbreviations

3D: 3-Dimensional
CT: Computerized tomography
PROMIS: Patient-Reported Outcomes Information System
PF: Physical function v1.2
PI: Pain interference v1.1
AP: Anteroposterior
NIH: National Institute of Health
CAT: Computerized adaptive testing
PRO: Patient-reported outcome
ORIF: Open reduction and internal fixation
PACS: Picture archiving and communication system
BMI: Body mass index
Add-Invert: Adduction-inversion
BMC: Bone marrow concentrate
*r*: Correlation coefficient
*p*: *p* value
*a*: Alpha value
MT: Metatarsal
PROMIS10: A global PROMIS (Patient-Reported Outcomes Information System) domain
VAS: Visual analog scale
FAAM: Foot and ankle ability measure
PASS: Patient acceptable symptom state
